# The First De Novo Transcriptome Assembly and Transcriptomic Dynamics of the Mangrove Tree *Rhizophora stylosa* Griff. (Rhizophoraceae)

**DOI:** 10.3390/ijms222111964

**Published:** 2021-11-04

**Authors:** Matin Miryeganeh, Hidetoshi Saze

**Affiliations:** Plant Epigenetics Unit, Okinawa Institute of Science and Technology Graduate University, 1919-1 Tancha, Onna-son, Okinawa 904-0412, Japan; hidetoshi.saze@oist.jp

**Keywords:** mangrove, RNA-Seq, gene expression, epigenetics, abiotic stress, salt stress, *Rhizophora stylosa*

## Abstract

Mangroves are salt-tolerant plant species that grow in coastal saline water and are adapted to harsh environmental conditions. In this study, we de novo assembled and functionally annotated the transcriptome of *Rhizophora stylosa*, the widely distributed mangrove from the largest mangrove family (Rhizophoraceae). The final transcriptome consists of 200,491 unigenes with an average length, and N50 of 912.7 and 1334 base pair, respectively. We then compared the genome-wide expression profiles between the two morphologically distinct natural populations of this species growing under different levels of salinity depending on their distance from the ocean. Among the 200,491 unigenes, 40,253 were identified as differentially expressed between the two populations, while 15,741 and 24,512 were up- and down-regulated, respectively. Functional annotation assigned thousands of upregulated genes in saline environment to the categories related to abiotic stresses such as response to salt-, osmotic-, and oxidative-stress. Validation of those genes may contribute to a better understanding of adaptation in mangroves species. This study reported, for the first time, the transcriptome of *R. stylosa*, and the dynamic of it in response to salt stress and provided a valuable resource for elucidation of the molecular mechanism underlying the salt stress response in mangroves and other plants that live under stress.

## 1. Introduction

Mangroves are adapted to harsh conditions such as high salinity, extreme tide, strong winds, intense heat, and anaerobic soil. They have developed specific characteristics, such as breathing- and support-roots, salt-excreting leaves, and viviparous seedlings in order to adjust to their stressful environment. Mangrove forests generally show gradual changes in their structure according to the level of stress in the environment. For example, they usually show shrub-like phenotype with smaller leaves and thinner trunks when they live closer to the seawater in high salinity condition, whereas they grow to be tall trees, with larger leaves and thicker trunks when they live in the estuary of rivers [1]. They are valuable coastal resources that support various marine ecosystems and protect the land from natural disasters such as tropical storms and tsunami waves. Unfortunately, due to exploitation and deforestation, these important trees are experiencing rapid loss. Furthermore, because of the ongoing climate change and anticipated sea level rise, studying mangroves appears to be a necessity so that strategies could be planned to protect these endangered plant species [2]. 

Salinity is one of the top and most challenging environmental stressors for plants, and it even leads to other chains of pressures such as osmotic and oxidative stress [3,4]. Many studies in different plant species have reported the changes in expression of salt tolerance genes in response to high salinity (e.g., [5,6,7,8]). As salt tolerant plants that encounter with constant daily fluctuation of salinity level caused by tidal oscillations, mangroves are a very good model system for studying gene expression changes under natural alteration of salt stress and may help to identify potential salt- and other abiotic-stress response genes [9,10]. Lira-Medeiros et al. [11] reported an association between phenology of a mangrove tree (*Laguncularia racemosa*) and DNA methylation, where trees growing in salt marsh environment were strikingly smaller than the trees growing next to a river in freshwater. A recent transcriptome study has reported differential expression of stress-responsive genes between two populations of *Bruguiera gymnorhiza* in their natural environment as well [8]. In addition, an epigenetic study of the same species showed DNA hypermethylation in transposable elements (TEs) in *B. gymnorhiza* plants experiencing high-salinity stress, and suggested suppression of TEs in mangroves under high salt stress [12]. Nevertheless, research about mangroves is still rare, and the molecular mechanisms underlying their high adaptability to coastal zone is not well understood, mostly because of unavailability of genome references for these species. To this date, among more than 80 mangrove species, only four of them have had their genome assembled [12,13,14,15]. De novo RNA sequencing (RNA-Seq) and transcriptome assembly is a helpful and compromising way to study transcriptional profile and gene expression of non-model plants, with no available reference genome [16]. Transcriptome expression analysis can help us to understand the molecular mechanisms behind stress tolerance in plants and their adaptation to difficult environmental conditions [17,18].

**Figure 1:**
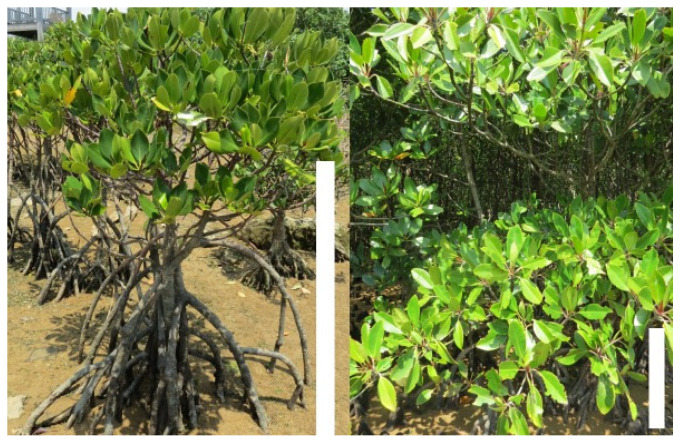
*R. stylosa* trees in the oceanside (**left**), and the riverside (**right**). Bars represent about one meter (m). Oceanside trees appear shorter (1–1.5 m) and the riverside trees grow to be as tall as 3–3.5 m.

The diversity of species in mangrove genera is often relatively low, having one or two species. This low diversity is believed to be related to harsh growth conditions of intertidal environments. The level of environmental stress plays an important role in defining the ecological boundaries for mangrove habitats. *Rhizophora* is known as the most diverse and dominant mangrove genus that grows at the front of mangroves, facing the sea with the ability of handling relatively high levels of salinity. *Rhizophora stylosa* is known to be best adapted to marine exposed offshore sites [19]. Here, we performed de novo transcriptome assembly, functional annotation, and differential transcriptome expression analysis of *R. stylosa*, to compare the genome-wide transcriptional profile between the two populations of this species growing under different levels of salinity in their natural habitat. The riverside population lives in the estuary of a river with the trees growing mostly in fresh/brackish water, and the oceanside population living in about 1 km apart in the shore of Pacific Ocean in saline water (Figure 1). The functional annotation of differentially expressed genes between the two populations categorized them mainly in the biological process of response to salt stress, response to osmotic stress, ion transport, and ROS scavenging. The transcriptome assembly provided here is a valuable resource for future research in mangroves and possibly in other tree species.

## 2. Results

### 2.1. Soil Salinity and Morphological Measurements

The average salinity level of the water surrounding the roots was recorded as 3.5% (ranging from 3.0% to 4.0%) at the oceanside, and 2% (ranging from 1.5% to 2.5%) at the riverside (Table S1). Morphological traits of 12 *R. stylosa* individual trees from both oceanside and riverside (six individuals from each side) were measured and recorded on the sampling day. We measured tree height, and tree diameter at breast height (DBH). In addition, 20 leaves from the distal end of lower branches of those 12 trees were collected to measure the mean leaf length, and leaf width. Mean height of the trees appeared to be 0.9 m for the oceanside trees, and 3.1 m for the riverside trees (Table S1). The mean tree DBH was measured to be 18.2 cm for the oceanside samples and 53.6 cm for the riverside samples. The mean leaf length was measured to be 8.8 cm for the oceanside trees, and 3.7 cm for the riverside trees. Mean leaf width was measured to be 2.8 cm for the oceanside trees, and 6.9 cm for the riverside trees (Table S1).

### 2.2. De Novo Assembly and Quality Assessment of the Transcriptome

Young leaves from the ten *R. stylosa* individual trees (six from the oceanside and four from the riverside), were collected for RNA-Seq that resulted in a total of ~74 giga base pair (Gb) of 150 base pair (bp) length paired-end reads (Table S2). After removing the adaptors, low quality sequences, and short reads, a total of ~66.5 Gb filtered, clean reads remained (Table S3). About 99.2%, and 97.8% of bases in clean reads were at the Q value of ≥20 and ≥30, respectively (Tables S2 and S3). The GC content was 41%. The initial assembly resulted from Trinity pipeline (version 2.4) (minimum contig length = 200 bp and minimum *kmer* covariance = 2) [20], was trimmed by removing the transcripts that were shorter than 300 bp, which resulted in a 267.5 mega base pair (Mbp) long transcriptome; (286,231 total transcripts with an average length of 934.6 bp, and N50 of 1381 bp) (Table S4). Redundant transcripts were then clustered using the program CD-HIT [21], and final assembly was ~183 Mbp long; (200,491 total transcripts with an average length of 912.7 bp and N50 of 1334 bp) (Table 1). The assembled transcripts ranged from the 300 bp cut-off value to the maximum length of 13,191 bp. Most of the final transcripts ranged from 300–500 and 501–1000 bp, and the frequency of longer transcripts gradually decreased (Figure S1), which is a common pattern reported for the transcriptome assemblies of other plant species as well [8,16].

To evaluate the quality of the final transcriptome, the filtered unique reads were mapped to the final assembly using Bowtie2 [22]. The overall alignment rate was >96%, where about 31% of the reads uniquely mapped to a single assembled transcript, and about 63% of the reads aligned to more than one transcript (Table S5). The completeness of the final assembly was further examined by the BUSCO (Benchmarking Universal Single-Copy Orthologs) algorithm through comparing them to the set of Embryophyta genes using BUSCO quality assessment tool [23]. As shown in Table 1, among the 1440 orthologous gene sets of Embryophyta, 90.8% (1308 BUSCOs) were “complete” BUSCO copies (including 504 (35.0%) single-copy and 804 (55.8%) duplicated), 4.5% (65 BUSCOs) were “fragmented”, and the remaining 4.7% (67 BUSCOs) were “missing” which shows the good quality of the assembled transcriptome. Organism distribution in the final transcriptome was searched through BLASTx and BLASTp, and *Arabidopsis thaliana* was identified as the species with the most homologous genes as *R. stylosa* that is consistent with the previous report for a close relative mangrove species, *B. gymnorhiza* [8] and different from what was reported for another *Rhizophora* species (*R. apiculata*) for which *Medicago truncatula* was assigned as the species with most homologous genes, however *Arabidopsis thaliana* was also found among the top species with high similarity [24] (Figures S2 and S3, Tables S6–S9).

### 2.3. Functional Annotation of the Transcriptome

Potential functions of the *R. stylosa* transcripts and the homologous genes of the predicted proteins were identified by following the Trinotate pipeline (available online: https://github.com/Trinotate/Trinotate.github.io, access date: 10 October 2019). Open reading frames (ORFs), and potential coding sequences were predicted using TransDecoder-3.0.1 [20]. The initial 200,491 non-redundant unique transcripts (unigenes) were predicted into 131,070 ORFs and 101,462 potential proteins (Table S10). The obtained nucleotide, and protein sequences were searched for the homologous nucleotides, and proteins through BLASTx and BLASTp, respectively (e-value < 1×10^−5^), using the UniProtKB/Swiss-Prot, and UniRef90 databases (Table S11). Furthermore, the known functional protein domains and potential signal peptides were searched using the Pfam protein domain database and SignalP database, respectively. In total, 119,851 nucleotide sequences out of the 200,491 transcripts (59.77%) and 75,491 protein sequences out of the 131,070 ORFs (57.6%) showed significant homology when aligned against the UniProtKB/Swiss-Prot database using BLASTx and BLASTp searches, respectively. A total of 59,197 (45.16%) unique Pfam protein motifs could be assigned, and 7044 (5.37%) protein sequences were predicted to have signal peptides (Tables S10–S12). Details of the transcriptome annotation, including Swiss-Prot, GO, KEGG and eggNOG mappings, PFAM predicted protein domains, signal peptides, and transmembrane domains can be found in the Table S11. In summary, the obtained de novo assembled transcriptome and comprehensive annotated transcripts of the mangrove tree *R. stylosa* are of good quality that makes them a very valuable resource for the molecular studies in mangroves and other trees that live under stress.

The detected transcripts were then related to GO and biological pathways using the DAVID Gene Ontology [25], and the REVIGO [26] web servers for further functional annotation. GO analysis of the BLASTx output, revealed 51,358 sequences associated with 884 GO terms. Among the three main categories, BP (Biological Process) category was the most abundant (17,722 sequences, 463 GOs), followed by CC (Cellular Component) (20,454 sequences, 204 GOs) and MF (Molecular Function) (13,182 sequences, 217 GOs) categories (Tables S13–S15). The GO analysis of BLASTp output revealed 47,913 sequences assigned to 804 GO terms. Among the three main categories, BP category was the most abundant (15,281 sequences, 432 GOs), followed by (CC: 20,450 sequences, 164 GOs) and (MF: 12,182 sequences, 208 GOs) categories (Tables S16 and S17). Within the BP category, response to salt stress, heat stress, osmotic stress, intense light, hypoxia, and response to biotic stresses such as bacteria and pathogen, were the most represented (Tables S13 and S16; Figures S4 and S5). In addition, genes in the category of ion transport were among the top represented. Interestingly, many epigenetic related BP categories such as genes encoding for DNA methylation, and histone modification, were among the enriched categories as well (Tables S13 and S16; Figures S4 and S5). Genes encoding for ATP-, protein-, DNA-, RNA-, and metal ion binding, were among the most abundant in the MF category. Some epigenetic categories such as methylated histone binding, and acetyltransferase activities were enriched in MF category as well (Tables S15 and S18; Figures S4 and S5). Among the CC categories, cytoplasm, plasma-, vacuole-, Golgi-, and chloroplast-membrane were the most abundant (Tables S14 and S17; Figures S4 and S5). Finally, to identify the functional biological pathways in *R. stylosa*, the detected proteins were mapped against the KEGG (Kyoto Encyclopedia of Genes and Genomes) database, which resulted in identifying 36 main metabolic pathways (Tables S19 and S20). Among them, biosynthesis of antibiotics, carbon metabolism, and plant–pathogen interaction were the most represented pathways (Figure S6).

### 2.4. Differential Expression Analysis and FUNCTIONAL annotation

**Figure 2:**
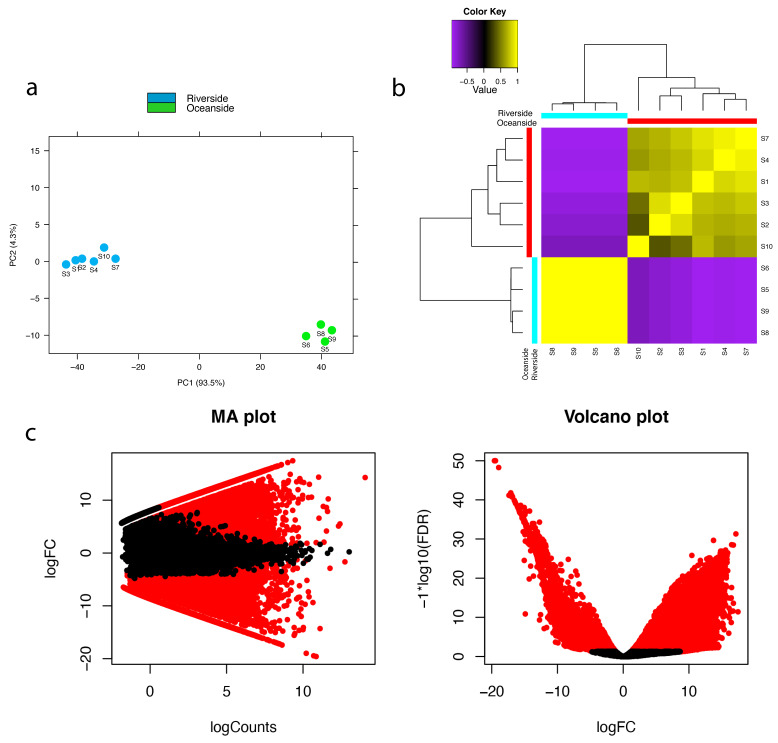
(**a**) Principal component analysis of transcript abundance (**b**) correlation matrix showing relationship between all samples as well as replicates. S1–S4 and S7, S10 are the six replicates from the riverside population and S5, S6, S8, and S9 are the four replicates from the oceanside population. (**c**) Pairwise comparisons of transcript abundance. MA plots showing average log fold change (logFC) vs average log of counts among oceanside vs. riverside transcripts across replicates. Volcano plots showing differentially expressed transcripts in relation to FDR (False discovery rate) for oceanside vs. riverside transcripts. Features found DE at FDR < 0.05 are colored red. Features with *p*-values at most 1 × 10^−3^ and at least 2^−2^-fold change are differentially expressed.

The correlation among replicates for the ten RNA-seq samples was examined using PCA analysis (Figure 2a) and correlation matrix (Figure 2b) which approved a high correlation. To investigate the differential expression of the unigenes in *R. stylosa* populations living in different levels of stress, transcript abundance was quantified using RSEM (RNA-Seq by Expectation-Maximization) [27]. The EdgeR (Empirical Analysis of Digital Gene Expression) package was used to extract differentially expressed genes (DEGs) at a minimum fold change of 2^2^ with the *p*-values at most 1 × 10^−3^. Among all the 200,491 unigenes, 40,253 were identified as differentially expressed (Table S21, Figure 2c) where 15,741 unigenes were upregulated (Table S22) and 24,512 were downregulated (Table S23) in the oceanside plants (saline condition).

**Figure 3:**
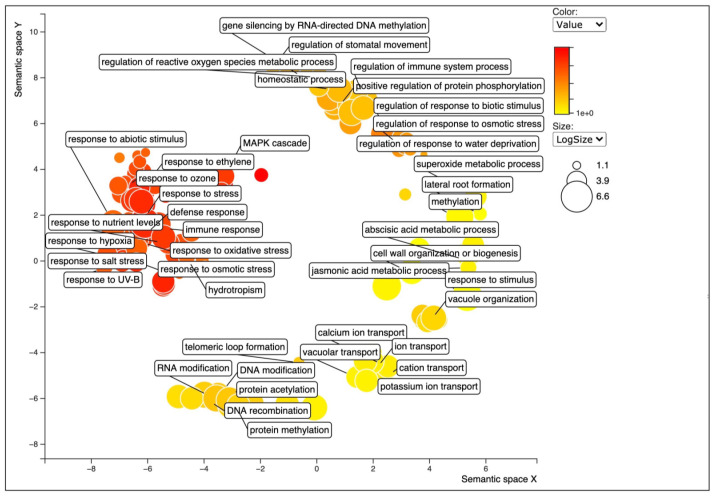
Biological process category of Gene Ontology (GO) enrichment analysis of upregulated transcripts of the oceanside trees, when compared with nr database using REVIGO. Circles in closer proximity have more closely related GO terms. The size of the circles indicates the number of GO terms. The color of the circle represents the significance of the enriched GO terms.

**Figure 4:**
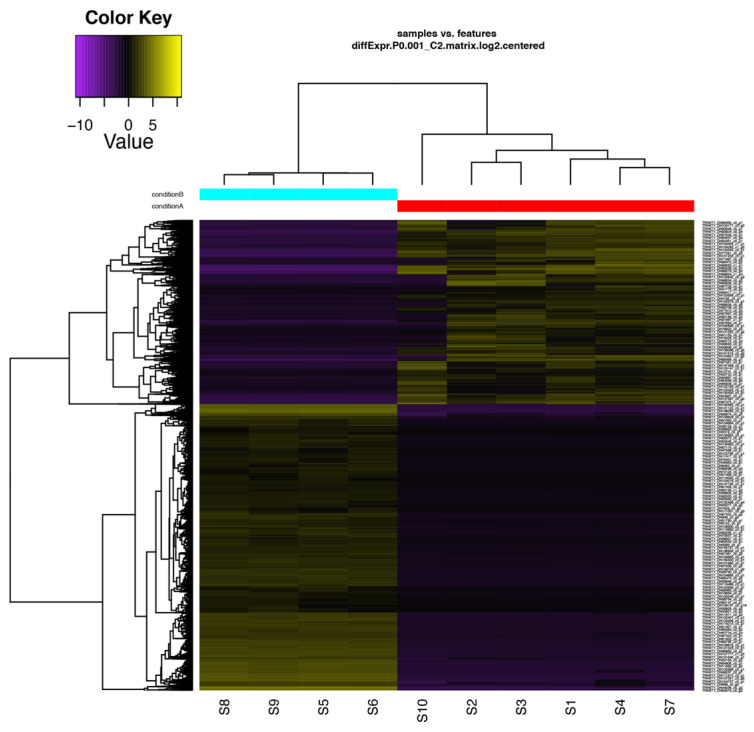
Hierarchical clustering of differentially expressed transcripts of oceanside-riverside *R. stylosa* leaf samples. Heatmap showing the relative expression levels of each transcript (rows) in each sample (columns). Rows and columns are hierarchically clustered. Expression values (FPKM) are log_2_-transformed and then median-centered by transcript. S1–S4 and S7, S10 are the six replicates from the riverside population and S5, S6, S8, and S9 are the four replicates from the oceanside population. Condition A and Condition B refer to the riverside and the oceanside, respectively.

GO enrichment analysis was performed to functionally annotate the DEGs (Tables S24–S29). Upregulated transcripts were grouped into 1799 GO terms. BP category was the most abundant (1181 GOs), followed by MF (396 GOs) and CC (222 GOs) categories (Tables S24–S26, Figure S7). In case of the downregulated transcripts, GO analysis revealed 2818 sequences associated with 115 GO terms. Among the three main categories, MF category was the most represented (21 GOs), followed by BP (18 GOs) and CC (5 GOs) categories (Tables S27–S29). The GO terms of these differential expressed genes reflected the higher level of stresses in the oceanside plants (Figure 3 and Figure S7). Among upregulated transcripts, within the BP category, response to stresses (including salt stress, light stimulus, osmotic stress, water deprivation, oxidative stress, nutrient level, UV), ion transport, oxidation reduction process, and homeostatic process were the most represented. Interestingly, many epigenetic related categories such as methylation, histone acetylation and histone methylation/demethylation, RNA directed DNA methylation (RdDM pathway related) genes, gene silencing, and siRNA involved in RNA interference were also represented (Figure 3 and Figure S7). The differentially expressed features with similar expression patterns were grouped into clusters (Figure 4 and Figure S8).

In the CC category, cellular component, cytoplasmic part, membrane-bounded organelle, nucleus, chloroplast, vacuole, Golgi, and cell wall were the most represented GO terms (Figure S7). Genes encoding catalytic activity, ion-, DNA-, RNA-, ATP-, and protein-binding were most abundant in the MF category. In addition, genes encoding proteins related to transporter activity, oxidoreductase activity, methyltransferase and acetyltransferase activity, heat-shock protein binding, and MAP Kinase activity were significantly enriched (Figure S7). Enrichment of these categories indicates the importance of stress response genes in *R. stylosa*. Differentially expressed genes were then subjected to KEGG annotation analyses and were clustered into 42 (21 upregulated and 21 downregulated) KEGG pathways (*p* < 0.05). Among the most enriched pathways were genetic information processing, carbohydrate metabolism, plant–pathogen interactions, MAPK signaling pathway, transport and catabolism, environmental information processing, alpha-linolenic acid metabolism, and lysine degradation (Tables S30 and S31, Figure S9).

### 2.5. Verification of RNA-Seq Data by qRT-PCR

**Figure 5:**
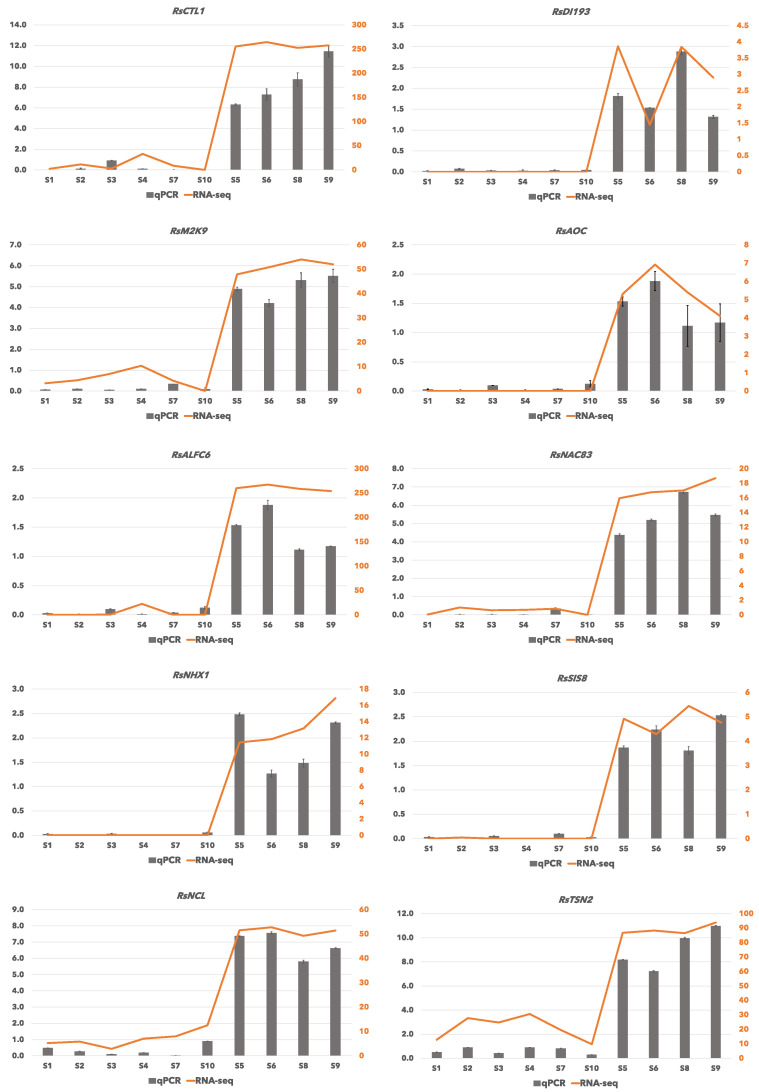
Verification of RNA-Seq results by real-time quantitative PCR. Log_2_ value of the gene expression in oceanside/riverside for 10 selected genes. RNA-Seq results -log fold change (logFC)- are shown as bars and qRT-PCR results are shown as lines for relative gene expression [log_2_ (Gene/ACT_2_)]. The 10 unigenes were named: *RsCTL1* (homolog of *AtCTL1*: Chitinase-like protein-1), *RsDI193* (homolog of *AtDI193*: Protein DEHYDRATION-INDUCED19-homolog-3), *RsM2K9* (homolog of *AtM2K9*: Mitogen-activated protein kinase kinase-9), *RsAOC* (homolog of *OsAOC*: Allene oxide cyclase), *RsALFC6* (homolog of *AtALFC6*: Fructose-bisphosphate aldolase-6), *RsNAC83* (homolog of *AtNAC83*: NAC domain-containing protein-83), *RsNHX1* (homolog of *AtNHX1*: Sodium/hydrogen exchanger-1), *RsSIS8* (homolog of *AtSIS8*: Probable serine/threonine-protein kinase-SIS8), *RsNCL* (homolog of *AtNCL*: Sodium/calcium exchanger NCL), and *RsTSN2* (homolog of *AtTSN2*: Ribonuclease TUDOR-2) (Table S32).

To validate the expression patterns revealed by RNA sequencing, quantitative real-time PCR (qRT-PCR) analysis was performed using aliquots of the same RNA samples. Ten upregulated unigenes that belonged to the GO categories of “response to salt and osmotic stress”, GO:0009651 and GO:0071470, respectively (Table S32), were chosen to be examined for their expression pattern among all 10 samples (six from the riverside and four from the ocean side) using qRT-PCR. The results of qRT-PCR were consistent with the RNA-Seq results and showed similar patterns of expression (Figure 5).

## 3. Discussion

Mangroves have evolved to live in harsh, dynamic conditions of soft, low oxygen soils, with high and fluctuating salinity levels in coastal regions. They provide nurseries for young sea creatures, and help to stabilize shorelines and prevent erosion, by protecting the land from high waves and storms. Despite their ecological importance, the available genomic and transcriptomic profiles for mangroves are very limited and the molecular mechanisms behind their extreme adaptability to stressful coastal zone is not well understood. Here we reported, for the first time, a de novo assembly and annotation of leaf transcriptome of a mangrove tree species, *R. stylosa* which is the most dominant genus of mangroves with the most diversity [19]. The number of the retrieved unitranscripts and unigenes, the N50, the average and the maximum length of the transcripts were all higher than what has been reported so far for other mangrove species. In addition, the GC content of the assembled unitranscripts (41.44%) was in the same range as the other mangroves [8,24]. The mapping rate of the RNA-Seq reads to the final transcriptome indicated a high quality for the assembly, with the overall alignment rate of >94.02%, covering 96.46% of the reference sequences (Table S5). The consistency of the final assembly was confirmed using BUSCO-3.0.2 as well (Table 1), where the quality of the *R. stylosa* transcriptome was comparable to, or better than those of most transcriptome assemblies listed in [28]. The transcriptome assembly contained more than 90% of genes represented at the available orthologue groups (Table 1). Furthermore, about 60% of the unitranscripts and 57.5% of the predicted proteins were assigned to genes, by searches through BLASTx and BLASTp against the UniProtKB/Swiss-Prot database (Table S10) which is similar to the previous mangrove study [8] and higher than that of other tree species such as Downy Oak (*Quercus pubescens*) [29].

BLAST search found the closest species with the most identical hits with *R. stylosa* (Top BLAST hits), to be *Arabidopsis thaliana*, rice, and tabaco (Figures S2 and S3, Tables S6–S9). In addition, many woody plants such as grape (*Vitis vinifera*), poplar trees (*Populus* spp.), rubber tree (*Hevea brasiliensis*), Cassava (*Manihot esculenta*), a mangrove tree (*Bruguiera gymnorhiza*), apple (*Malus domestica*), apricot (*Prunus armeniaca*), coffee (*Coffea arabica*), melon/orange/tangerine (*Citrus* spp.), cherry (*Prunus avium*), peach (*Prunus persica*), kiwi (*Actinidia deliciosa*), *Eucalyptus globulus*, and olive (*Olea europaea*), also showed high similarity (with over 70% identical match). Interestingly, the leafy spurge (*Euphorbia esula*), which is known to have an exceptional resistance to conditions associated with oxidative stress in plants [30], was among the highly similar species with *R. stylosa* as well. These results are highly consistent with the previous study of *B. gymnorhiza* (Figure S10) [8]. However, the homologous hits found between the top species and the mangrove species in this study, *R. stylosa*, were higher than that of the previously reported mangrove, *B. gymnorhiza* (Figure S10). This could be either because of higher similarities between *R. stylosa* transcriptome and those spices, or simply because of a more complete transcriptome assembly and annotation in the current study. The latter seems to be more likely as *B. gymnorhiza* itself shared higher number of homologous hits in this study with *R. stylosa* (53 hits) as opposed to only seven hits in the previous report (Figure S10).

A recent transcriptome study of selected species from the family Rhizophoraceae, reported homologous BLAST hits between the non-mangrove member of the family, “*Carallia brachiate*” and *Lotus japonicus* coding genes [24]. Interestingly the non-mangrove member, *C. brachiate*, has developed aerial stilt roots, same as the only mangrove genus with this type of roots, i.e., *Rhizophora* [31]. In the current study, we found homology between *Rhizophora stylosa* and *Lotus japonicus* as well, which may imply that besides morphological similarity of having stilt roots, these two species may share deeper molecular connections which is worth to be investigated in future studies. In our previous study, *B. gymnorhiza* also shared homologous BLAST hits with *L. japonicus* that is suspected to link with its basal location in the phylogenetic tree of Rhizophoraceae [8]. *B. gymnorhiza* is the oldest mangrove species in the family Rhizophoraceae that has first diverged from the non-mangrove members and therefore it is expected for this species to share more similarity with non-mangroves [13,32].

An interesting finding in this study was the upregulation of many unigenes in the oceanside population that belonged to the category of epigenetic regulations such as DNA methylation (GO:0006304) and histone modification (GO:0016570) (Table S32). Future research on epigenetic regulations of stress tolerance genes in mangroves is necessary to investigate how epigenetics may affect the adaptation of mangrove species. In our recent study we reported DNA hypermethylation of transposable elements (TEs) in *B. gymnorhiza* which was associated with the expression of chromatin modifier genes and suggested the epigenetic regulation of TEs in this mangrove species under salt stress [12]. Previous transcriptome analysis of *B. gymnorhiza* has also reported a few differentially expressed epigenetic genes [8], although the number of epigenetic genes found in the current study for *R. stylosa* was significantly higher, which emphasizes the need for further investigation.

When plants experience stress, the expression of the genes that are involved in complex stress-response networks will change. High salinity is one of the main stress factors for plants where extra Na^+^ ions reduce the osmotic potential of surrounding soil, and the water exchange between roots and soil will become difficult, which then causes osmotic stress [33]. Response to osmotic stress is one of the main skills plants have developed to cope with salt stress, which is also connected to their other stress-responsive skills such as ion transport, ROS scavenging, and cell signaling [34,35]. Our GO enrichment analyses found genes belonging to these categories. The upregulated unigenes that were annotated within the biological process of “response to salt stress” and “hyperosmotic salinity stress” (gene categories of “GO:0009651” and “GO:0042538”, respectively) (Tables S13 and S24), were found to be homologous with genes encoding proteins that contribute to salt- and drought-tolerance by preventing the overaccumulation of sodium ions, and are involved in cytoplasmic Na^+^ detoxification in high salinity conditions (Table S32). 

Another important skill that plants have acquired in order to cope with high salinity stress is homeostasis and ion transport. High concentrations of Na^+^ in the vacuoles cause osmotic stress and to survive that, plants need to adjust the amount of ion transfer through their membrane system by controlling ion homeostasis and the excretion and intracellular segregation of excessive salt ions. Ion transporters in plants are embedded in their membrane system and are involved in several signaling pathways [36]. In our study, we found unigenes annotated in the BP categories of “ion transport” and “homeostasis” that were upregulated in the oceanside and were homologous with genes involved in K^+^ homeostasis and osmotic adjustment that confer tolerance to low potassium and high sodium conditions (Table S32). The upregulation of these genes under salt stress has recently been reported in a poplar tree (*Populus wutunensis*) as well [5]. Similarly, transcriptome analysis of a salt tolerant crop species (*Citrus limonia*), and a mangrove tree species (*B. gymnorhiza*) reported upregulation of salinity-responsive genes belonging to the BP categories of salt stress, osmotic stress, ion transport, and ion homeostasis as well [7,8].

Another life-threatening cause of damage for plants, resulting from high salinity stress, is the accumulation of excessive reactive oxygen species (ROS), that results in oxidative stress, and disturbs normal metabolism in cells, and may even result in death [37,38]. Plants adjust the regulation of certain genes in response to oxidative stress to arrange discarding the intracellular ROS, and therefore minimize the oxidative damage caused by high salt stress [39]. Recent transcriptome analyses reported the gene expression variation in *Populus wutunensis* and *B. gymnorhiza* under high salinity stress and the upregulated genes were found to be involved in ion transport, osmotic regulation, and reactive oxygen species (ROS) scavenging [5,8]. In the present study, the *R. stylosa* transcriptome analysis identified upregulation of many unigenes in the oceanside plants that were annotated into the BP category of “response to oxidative stress: (GO:0006979)”. These unigenes were homologues of genes that are known to contribute to oxidative stress tolerance (Table S32). A transcriptome study of a salt tolerant cotton variety (*Gossypium hirsutum*) has reported differentially expressed genes from the BP category of “response to oxidative stress” as well [40]. Another gene expression study in a salt tolerant plant (*Suaeda salsa*) also has shown differentially expressed genes involved in the BP category of reactive oxygen species (ROS) scavenging [41].

Among the other upregulated unigenes found in this study were those belonging to the gene category of “lignin metabolic process” (GO:0009808) and “cellulose biosynthetic process” (GO:0030244). Cellulose and lignin are materials for secondary walls in plants and make a critical protection mechanism against biotic stresses. The homologous genes of those unigenes are known to encode for proteins that guard the plant against pathogens (Table S32). Pathogens have been suggested to be one of the main biotic stressors for mangrove trees in their natural habitats [8,12] which is also seen in other plant species such as cotton (*Gossypium hirsutum*) [8,40]. Furthermore, large families of MAPK (mitogen-activated protein kinase) pathway components in plants are known to be activated in response to stressors such as salt, drought, heat, and wounding [42]. In our study, genes from the MAPK signaling pathway were found to be upregulated as well (Tables S24 and S32). The presence of transcripts homologous with the above-mentioned genes provides new insights into molecular mechanisms behind high adaptability of *R. stylosa* and other mangroves to the harsh coastal habitats.

In summary, this study provided the first transcriptome assembly, and the first transcriptional dynamic of the species *R. stylosa* that has the widest distribution range among mangrove species. The differential gene expression analysis revealed the influence of salinity level on gene expression, which allowed us to identify and annotate genes associated with salt stress, osmotic regulation, ion transport, and oxidative stress. In addition, many genes involved in DNA methylation and histone modification were detected that suggest the possible role of epigenetic regulations in adaptation of mangroves. Based on our transcriptome results, it seems that mangrove plants use various mechanisms in response to their stressful environment and our results provide a good foundation to design future approaches to reveal those mechanisms. The identified pathways and set of candidate genes in this study are valuable assets that can facilitate the analyses of gene expression profiles related to salt tolerance in plants and serve as a good resource for future functional genomics to develop novel strategies for stress management in plants.

## 4. Materials and Methods

### 4.1. Plant Material, Soil Salinity, and Morphological Measurements

Young leaves from each of the 10 adult individuals of *R. stylosa* trees (four and six biological replicates from the oceanside and the riverside, respectively) in the mangrove forest located along the estuary of the Okukubi River, Okinawa Island—Japan, and the nearby coastal area of Pacific Ocean—Japan (26°27’ N, 127°56’ E) were collected, snap-frozen in liquid nitrogen, and stored at −80 °C for RNA extraction. The riverside population grew near the estuary of the river, and the oceanside population grew in less than one kilometer away in the shore of the Pacific Ocean—Japan. The sampling was performed from 12:30–13:00 on 28 Jun 2018, (temperature: 30 °C—humidity level 93%, precipitation: 0%). The salinity of the soil water surrounding the roots was measured using a Refractometer W/ATC 300011, SPER Scientific (Scottsdale, AZ, USA). The salinity was also measured every fortnight for one year between 12:30–13:00 to obtain the average salinity level in the field (Table S1). The morphological traits were measured using 12 randomly chosen *R. stylosa* individuals (six from each population). Tree height, and tree diameter at breast height (DBH) were measured. In addition, 10 leaves from the distal end of lower branches of those 12 trees were collected to measure the average leaf length and leaf width. The results were tested for significant differences between sites using the Welch Two Sample *t*-test in R software (http://www.R-project.org—version 3.5.0—April 2018) (Table S1).

### 4.2. RNA-Seq and Transcription Analyses

#### 4.2.1. RNA Extraction

A total of ten Hi-Seq libraries of RNA samples (four and six from the oceanside and the riverside, respectively) were sequenced. Total RNA isolation was performed using the method described in [43] combining a CTAB-based lysis solution with silica column-based RNA binding. RNA concentration was determined using Qubit (Qubit QC, Thermo Fisher Scientific, Waltham, MA, USA). The integrity of RNA samples was evaluated using an Agilent 2100 Bioanalyzer (Agilent Technologies- (Agilent Technologies Canada, Inc., Mississauga, ON, Canada)), and high-quality RNA samples (the integrity number ≥ 8.0) were used for library construction.

#### 4.2.2. Library Construction and Sequencing

The cDNA library for RNA-Seq was constructed using TruSeq™ RNA Library Prep Kit (Illumina, San Diego, CA, USA) according to the manufacturer’s instructions, and was sequenced using Next Seq (Illumina Inc., San Diego, CA, USA). Raw reads were trimmed using Trimmomatic-0.36 [44] with default parameter. Quality control of RNA-Seq reads, before and after trimming, was performed using FastQC-v0.11.3 (http://www.bioinformatics.babraham.ac.uk/projects/fastqc/, access date: 10 October 2019). All the Illumina sequencing reads generated in this study are deposited at the NCBI PRJNA769465.

#### 4.2.3. De Novo Transcriptome Assembly

Filtered, clean reads were used for transcriptome assembly using Trinity pipeline (version 2.4) with the default parameters [20]. Clustering of redundant transcripts was performed with 95% identity using CD-HIT-v4.6.4 [21]. Completeness of the final assembly was evaluated using BUSCO-3.0.2 analysis [23]. Furthermore, to estimate the consistency of the assembly and alignment rate, the filtered unique reads were mapped to the assembled transcriptome using Bowtie2 (https://sourceforge.net/projects/bowtie-bio/, access date: 10 October 2019).

### 4.3. Functional Annotation of the Transcriptome

Potential coding sequences and open reading frames (ORFs) were identified using TransDecoder-3.0.1 with default parameters [20]. Trinotate pipeline (available online at: http://trinotate.github.io, access date: 10 October 2019) was used for functional annotation [20]. Nucleotide transcripts and protein sequences were searched against the UniProtKB/Swiss-Prot and UniRef90 databases using NCBI-BLASTx and BLASTp v2.2.28+ (-evalue 1 × 10^−5^ -max_target_seqs 1 -outfmt 6), respectively. Predicted proteins were annotated using profile hidden Markov models with HMMER (v3.1b2) [45] against Pfam-A databases [46]. Based on these annotations, Gene Ontology (GO), Pfam and Kyoto Encyclopedia of Genes and Genomes (KEGG) terms were assigned to each unigene. In addition, prediction for signal peptides, transmembrane domains and rRNA transcripts was conducted by SignalP (v4.1) [47], TMHMM (v2.0) [48] and RNAMMER (v1.2) [49], respectively. The final annotation report was generated by loading all the above annotations into the Trinotate SQLite database.

### 4.4. Transcript Abundance and Differential Expression Analysis

The transcript abundance was quantified using the alignment-based methods and using the align_and_estimate_abundance Perl script built in the Trinity package. In this analysis, RSEM [27] was used as the abundance estimation method and bowtie2 (https://sourceforge.net/projects/bowtie-bio/, access date: 10 October 2019) was used as the alignment method. Gene Expression Matrix was built using the abundance_estimates_to_matrix.pl script. PtR script was used to generate correlation matrix and Principal Component Analysis (PCA) plot. Differentially expressed genes (DEGs) from the count matrix were found by using the EdgeR statistical package using the run_DE_analysis.pl script [50]. The analyze_diff_expr.pl script was used to examine GO enrichment. The differentially expressed (DE) features were partitioned into clusters with similar expression patterns by define_clusters_by_cutting_tree.pl script with Ptree method. The normalization factors were calculated using trimmed mean of M-values (TMM) method. The threshold FDR < 0.05 was adjusted to identify the differentially expressed genes by fold change (≥2). In addition, David Gene Ontology was used for functional annotation of expressed homologous gene pairs. The protein sequences were also searched against the KEGG database for KEGG Orthology (KO) assignments and pathway annotation. GO enrichment sets were further summarized using ReviGO (http://revigo.irb.hr/, access date: 10 October 2019) [26].

### 4.5. Validation of Differentially Expressed Genes by qRT-PCR

The differentially expressed genes that were overexpressed in the BP category of “response to salt and osmotic stress”, were searched through BLAST against nucleotide databases and were assigned to their homologous genes. Among them, ten genes were randomly selected as representatives to validate the data generated through RNA-Seq. The primer pairs for qRT PCR were designed by Primer3 [51,52] using the unigene sequences obtained in this study. The qRT-PCR was carried out using aliquots of the same RNA samples that were used for RNA sequencing using a Thermocycler BioRad-USA qPCR machine. Two micrograms (2 μg) of total RNA were used for cDNA synthesis by Prime Script II reverse transcriptase (TAKARA- Bio Inc. Japan) using an oligo(dT) primer. *ACT2* gene was used as internal control for normalization. The cDNA was diluted 5 to 10-fold, and the qPCR reactions were carried out with two technical replicates, using Takara SYBR Premix Ex Taq II (Takara Bio Inc. Japan) and incubated at 95 °C for 3 min followed by 40 cycles of 95 °C for 15 s, 58 °C for 15 s and 72 °C for 15 s. The primer sequences for the unigenes are provided in Table S33. PCR specificity was evaluated using melting curve analysis, and the expression levels were calculated using the 2^−∆∆Ct^ method [53]. Data were analyzed and plotted using Microsoft Excel 2010.

## Figures and Tables

**Table 1:** Summary of quality assessment of the transcriptome assembly of *R. stylosa* and BUSCO analysis results.

Quality Items and BUSCO Categories	Characteristics
Number of transcripts	200,491
Sum length (bp)	182,997,418
Minimum length (bp)	300
Average length (bp)	912.7
Maximum length (bp)	13,191
N50	1,334
%GC	41.44
Complete BUSCOs	1308 (90.8%)
Complete and single-copy BUSCOs	504 (35.0%)
Complete and duplicated BUSCOs	804 (55.8%)
Fragmented BUSCOs	65 (4.5%)
Missing BUSCOs	67 (4.7%)
Total BUSCO groups searched	1440

## Data Availability

All the Illumina sequencing reads generated in this study are deposited in the NCBI PRJNA769465.

## Supplementary Materials

The following are available online at https://www.mdpi.com/article/10.3390/ijms222111964/s1.
