# Supplementary material for: The First De Novo Transcriptome Assembly and Transcriptomic Dynamics of the Mangrove Tree Rhizophora stylosa Griff. (Rhizophoraceae)
Source: Int J Mol Sci. 2021 Nov 4;22(21):11964. doi: 10.3390/ijms222111964 (PMC8584393; doi:10.3390/ijms222111964)

Figure S1: Sequence length distribution of non-redundant (NR) unique unitranscript sequences after sequencing and de novo assembly of *R. stylosa* leaf transcriptome. The X-axis represents the length range bins in bp. The Y-axis represents the frequency of transcripts in each bin.

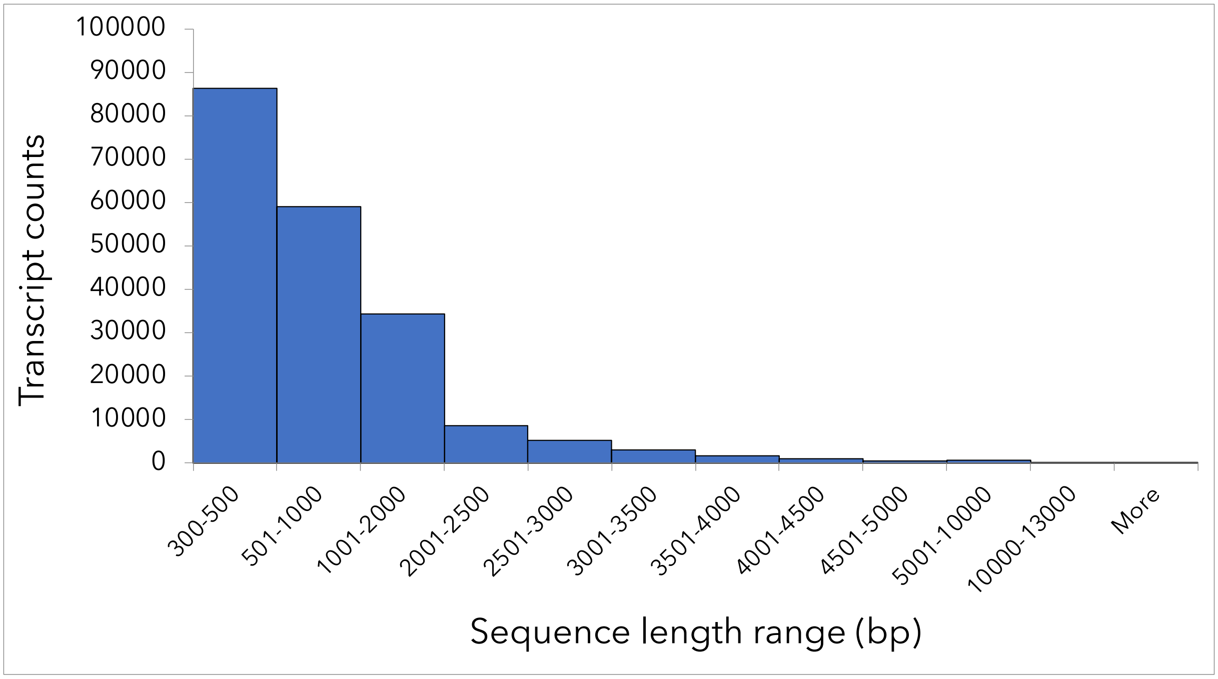

Figure S2. BLAST Top-Hits species distribution for *R. stylosa* leaf transcriptome when compared with nr database (BLASTx).

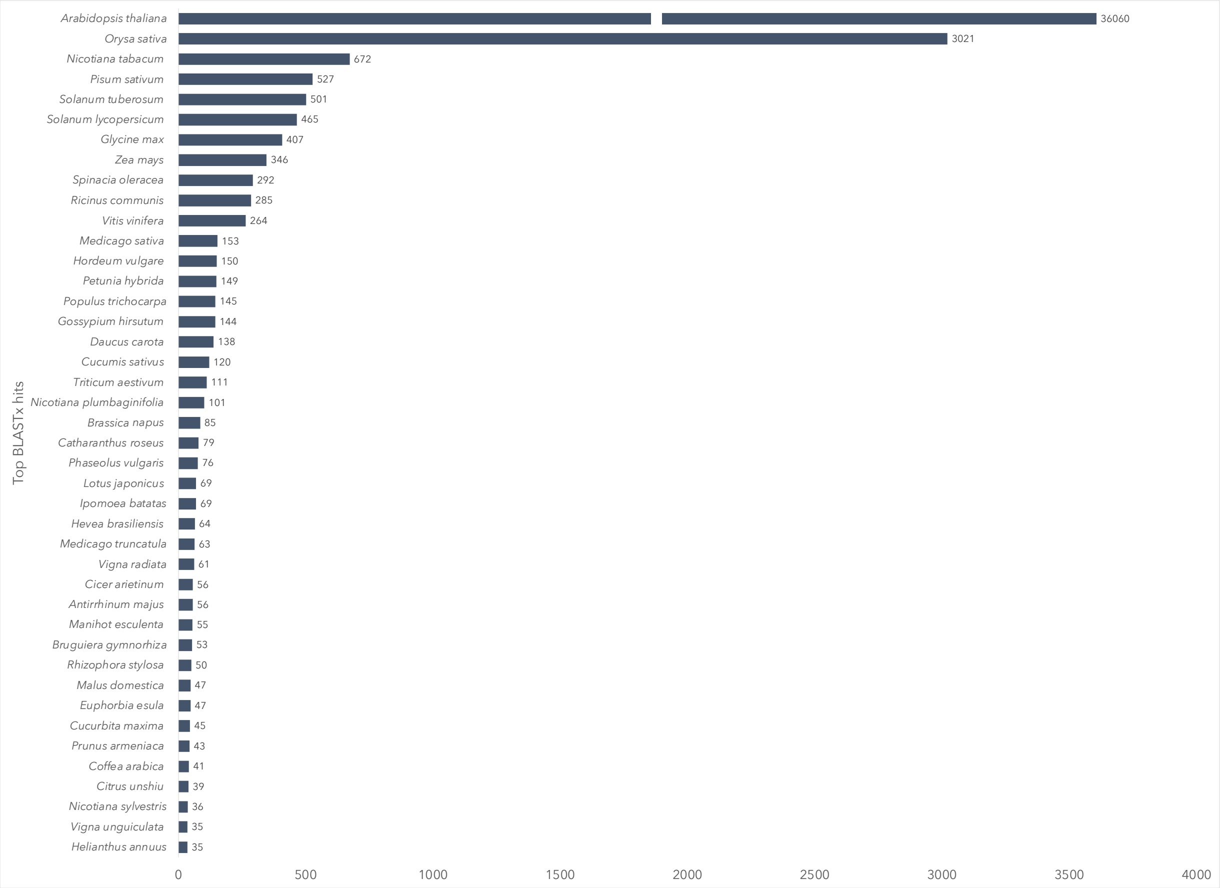

Figure S3. BLAST Top-Hits species distribution for *R. stylosa* leaf transcriptome when compared with Swiss-Prot database (BLASTp).

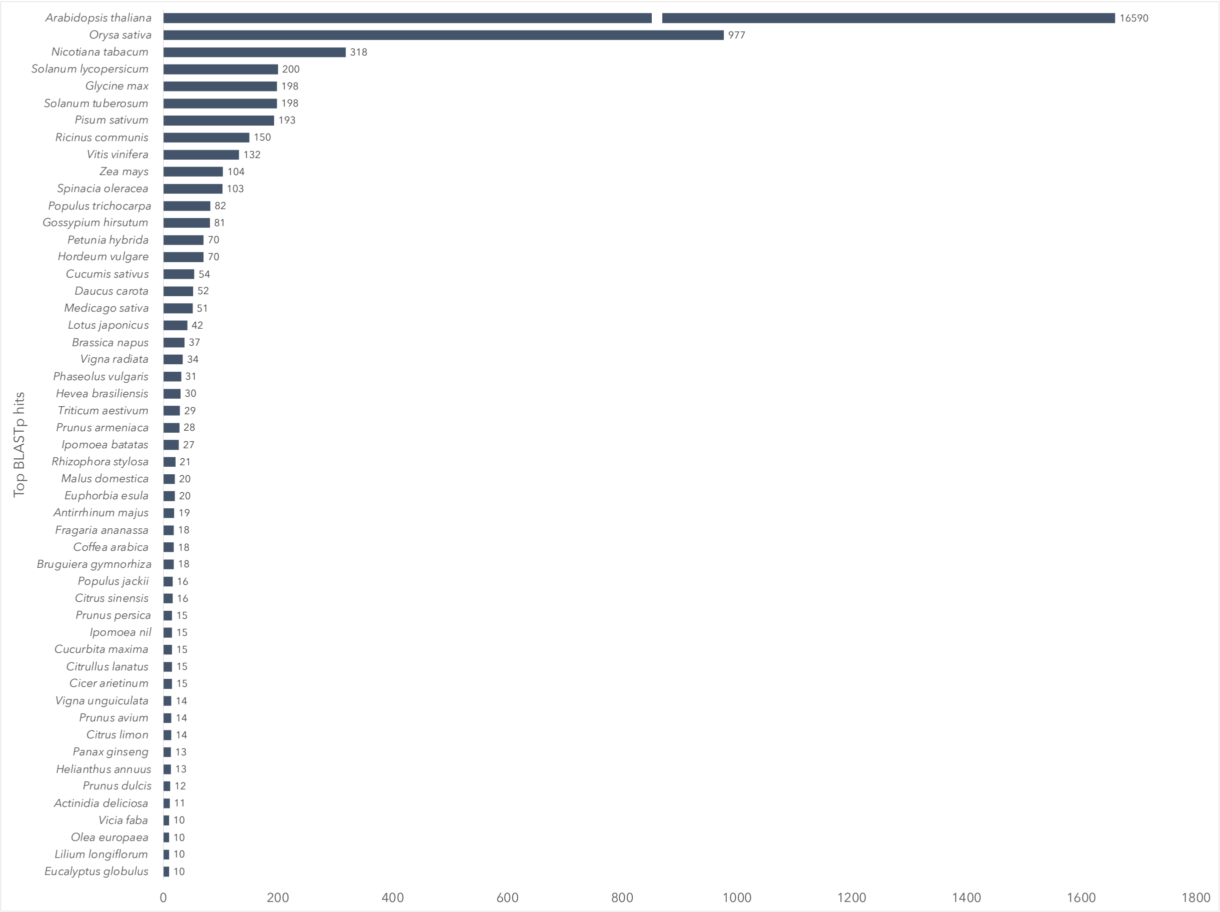

Figure S4. Gene ontology (GO) functional classification of *R. stylosa* leaf transcriptome BLAST Top-Hits species distribution when compared with nr database. Histograms of the frequency of transcripts annotated to specific GO categories; biological process, cellular components, and molecular functions are represented by blue, green, and orange bars, respectively.

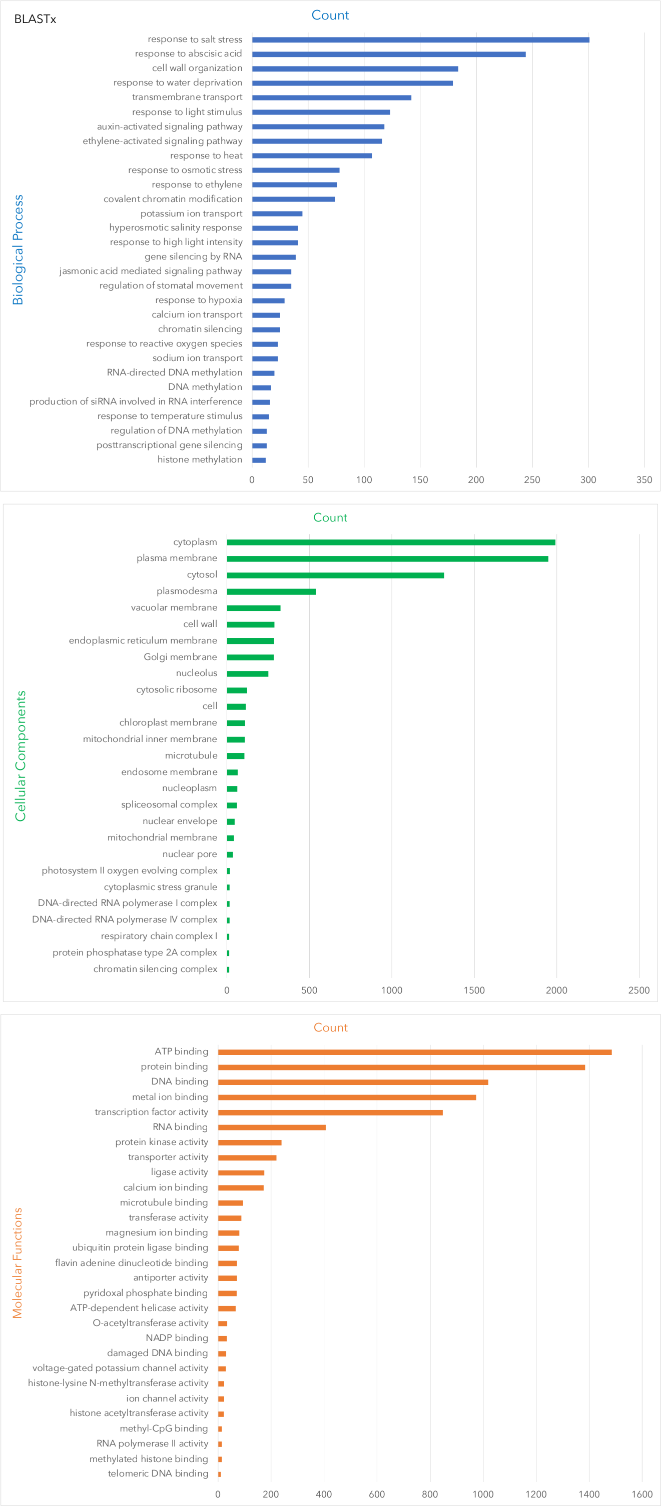

Figure S5. Gene ontology (GO) functional classification of *R. stylosa* leaf transcriptome BLAST Top-Hits species distribution when compared with Swiss-Prot database. Histograms of the frequency of transcripts annotated to specific GO categories; biological process, cellular components, and molecular functions are represented by blue, green, and orange bars, respectively.

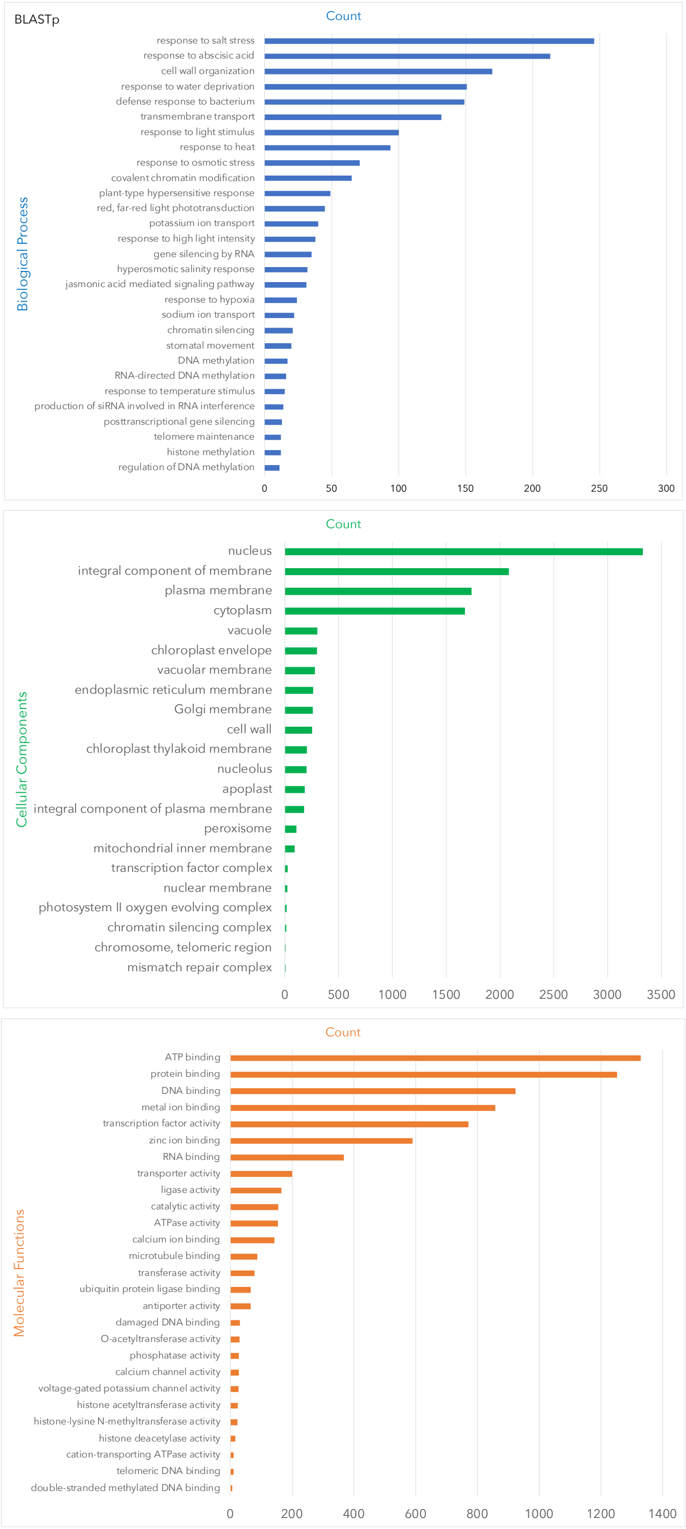

Figure S6. Kyoto Encyclopedia of Genes and Genomes (KEGG) analysis of *R. stylosa* leaf transcriptome BLAST Top-Hits species distribution when compared with nr database (top) and Swiss-Prot database (bottom). Histograms of the frequency of transcripts annotated to KEGG pathways.

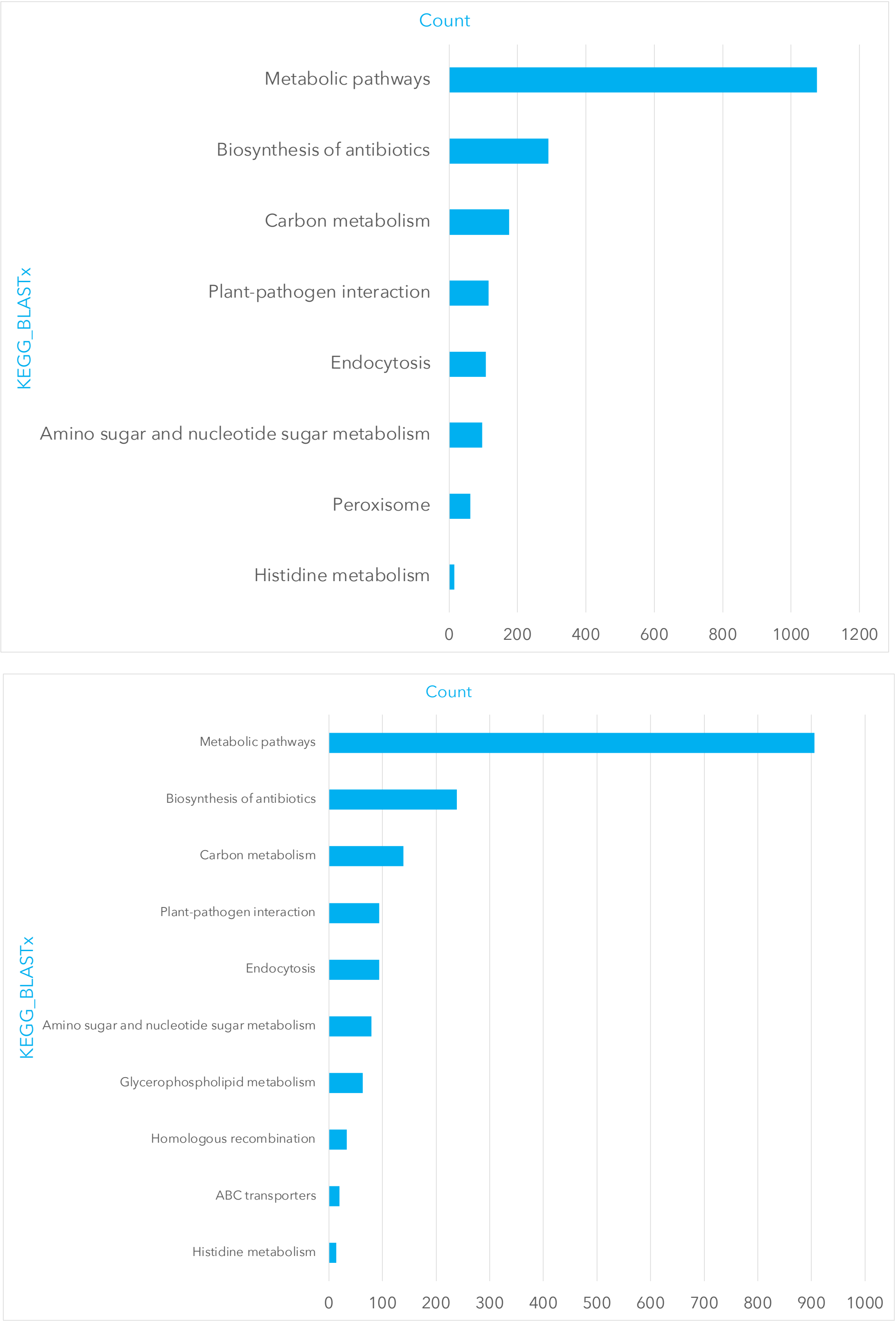

Figure S7. Biological process category of Gene ontology (GO) functional classification of upregulated transcripts BLAST Top-Hits species distribution when compared with Swiss-Prot database. Histograms of the frequency of transcripts annotated to specific GO categories; biological process, cellular components, and molecular functions are represented by blue, green and orange bars, respectively.

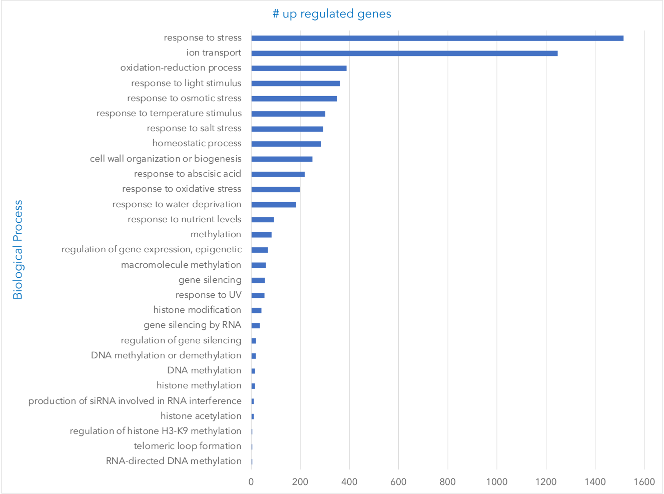

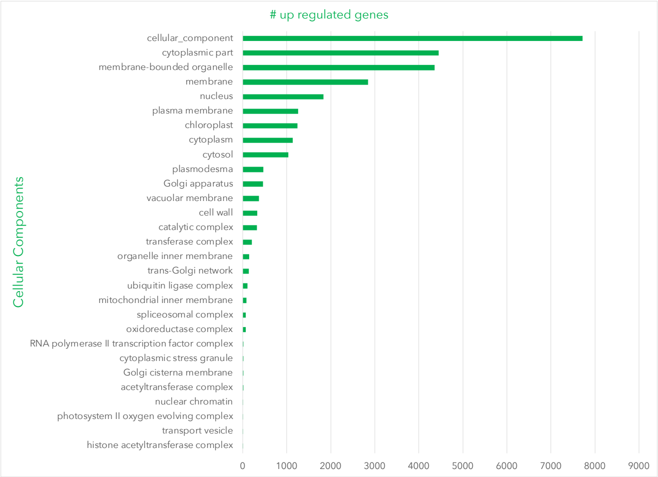

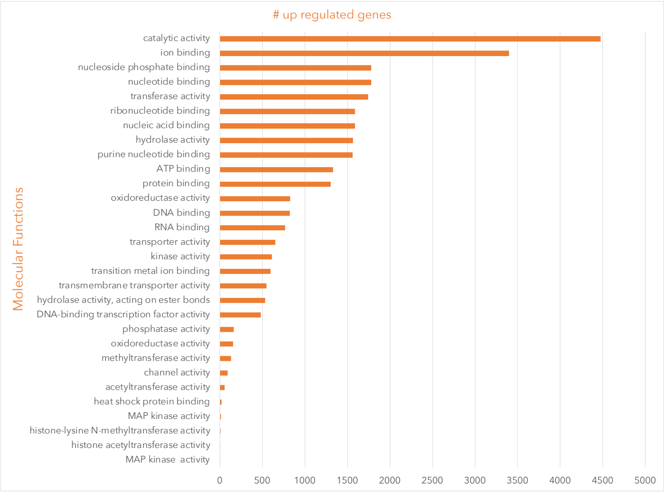

Figure S8. Transcript clusters extracted from the hierarchical clustering with R. X-axis: samples; y-axis: median-cantered log2(FPKM). Grey lines, individual transcripts; blue line, average expression values per cluster.

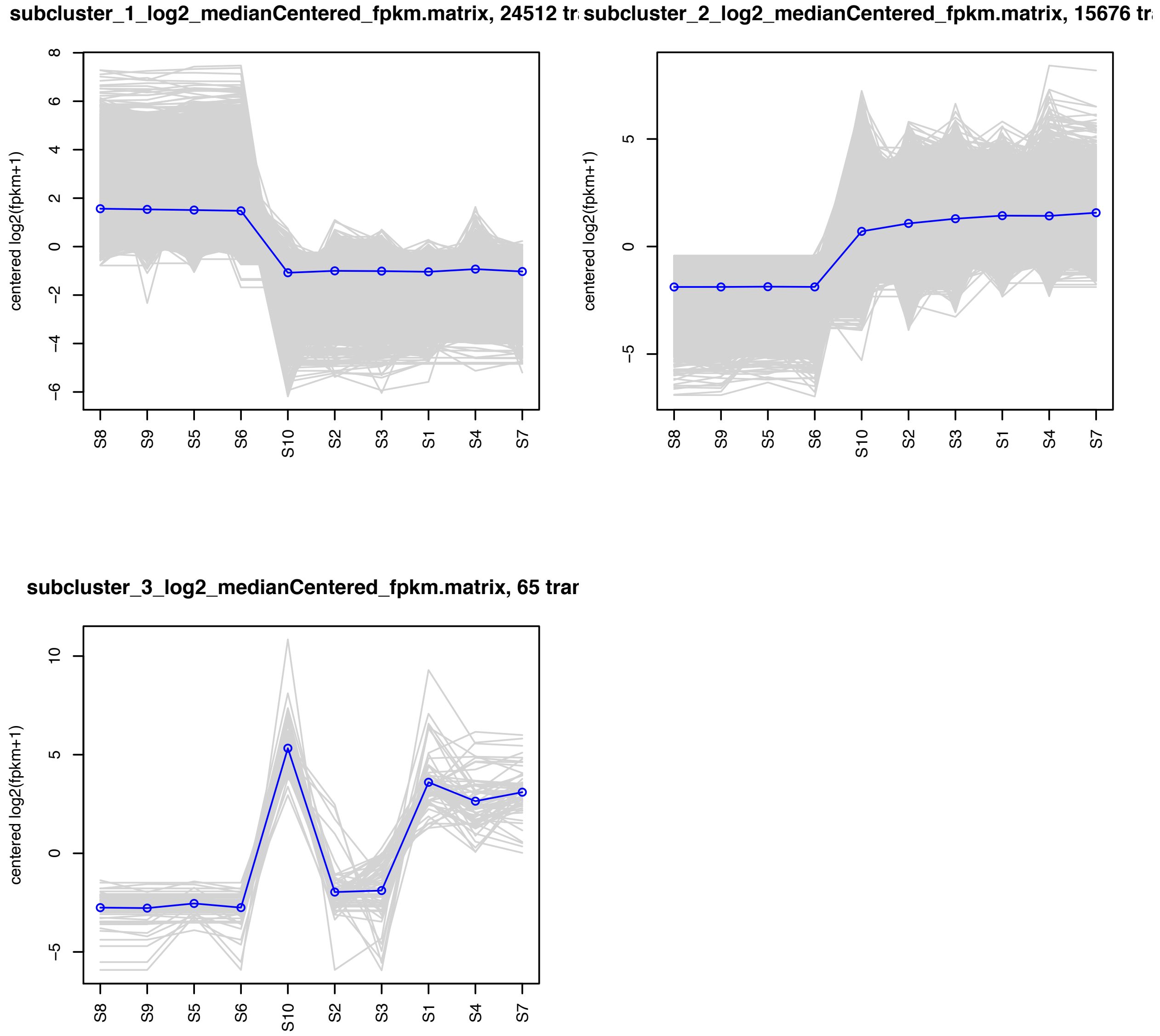

Figure S9. Kyoto Encyclopedia of Genes and Genomes (KEGG) analysis of upregulated (Top) and downregulated (bottom) transcripts in oceanside samples of *R. stylosa*.

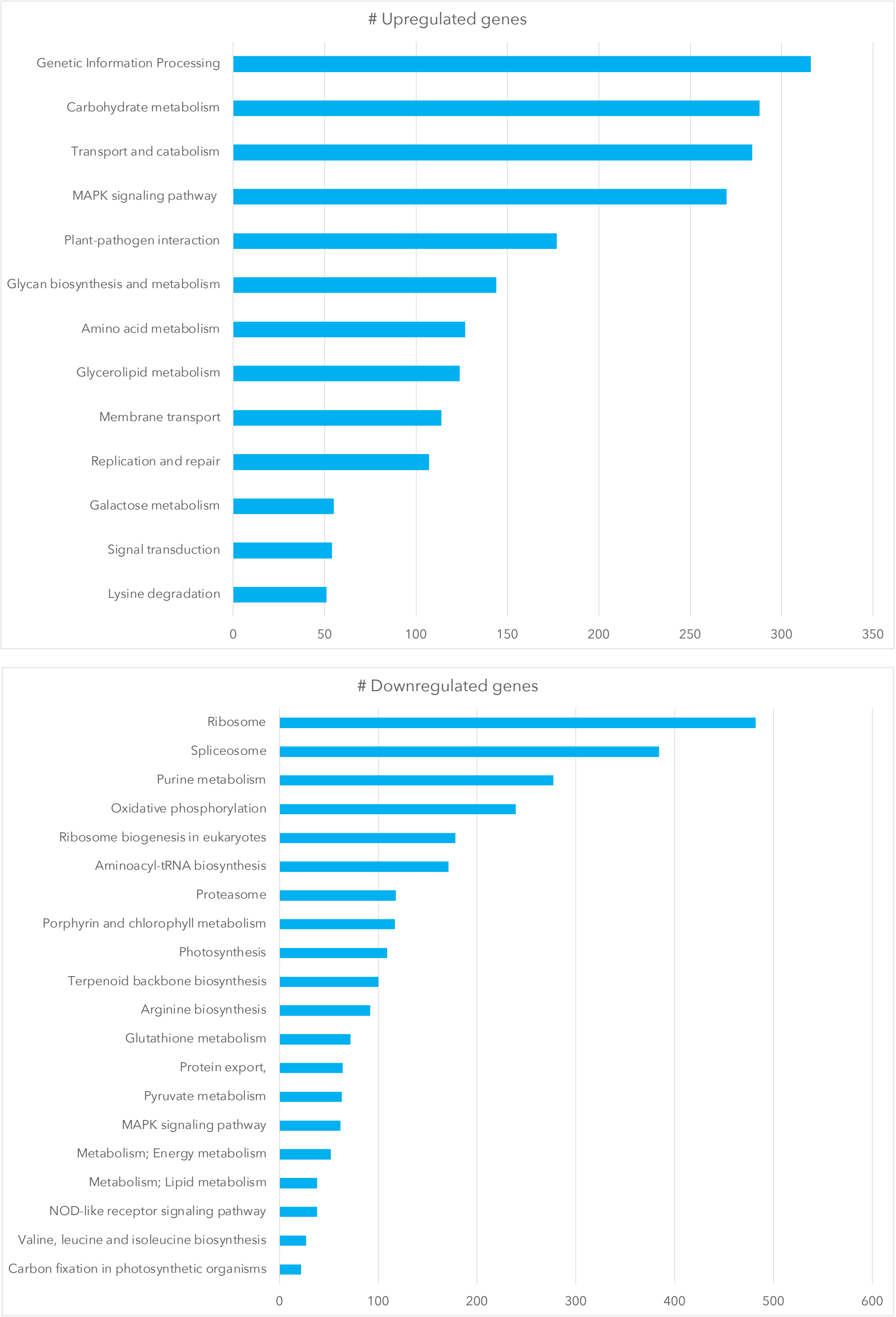

Figure S10. Comparison of BLAST Top-Hits species distribution for *R. stylosa* (green) and *B. gymnorhiza* (orange) leaf transcriptome when compared with nr database (BLASTx).

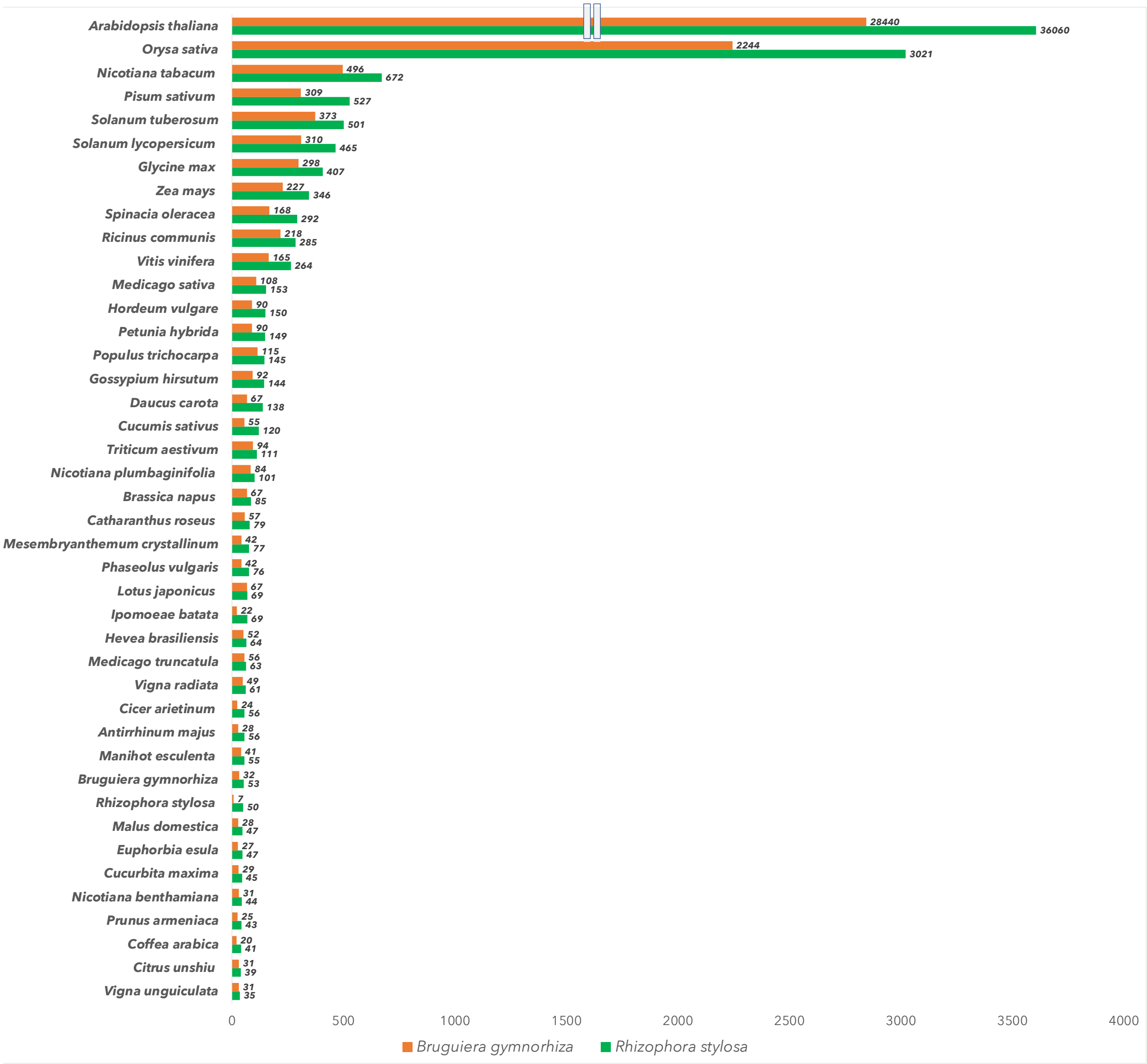

Supplement: Supplementary file 1 [file ijms-22-11964-s001.zip › Supplementary_Figures.pdf]
